# MEDICAL STUDENT EXPERIENCES WITH PELVIC EXAMS UNDER ANESTHESIA: A SCOPING REVIEW

**DOI:** 10.51894/001c.123053

**Published:** 2024-08-30

**Authors:** Vasha Reddy

**Affiliations:** 1 College of Osteopathic Medicine Michigan State University

## 68

### INTRODUCTION

Learning to perform pelvic examinations is vital to quality care, and medical Students practicing with an anesthetized patient may offer advantages due to relaxed pelvic muscles. However, concerns have arisen surrounding explicit informed consent for medical Students to be involved in this practice. This study aims to explore the attitudes and experiences of medical Students regarding pelvic exams under anesthesia (PEUA) and informed consent.

### OBJECTIVE

Identify the depth of existing literature on pelvic exams under anesthesia performed by medical Students without informed consent and medical Students’ attitudes toward this topic.

### METHODS

PubMed, Science Direct, and Web of Science were searched in December 2022 with using key search terms: including “pelvic exams,” “vaginal exams,” “gynecolog*,” “Students” “trainees,” “consent,” “permission,” “anesthesia,” “surgery,” and “operat*.” This yielded 227 articles. After deduplication, abstract screening, full text review, quality appraisal, and citation searching, six studies were analyzed. Inclusion criteria included publication date after 2012, location in English-speaking countries, and Student-reported data. Studies were excluded if they were opinion articles or commentary about gynecology education or informed consent in general.

### RESULTS

Among the six studies, Students in three studies had conducted a PEUA without consent. Three studies described circumstances of superiors directing Students to conduct a PEUA with unclear consent status. Four studies highlighted Students’ fear of receiving a poor evaluation if they were to refuse performing a PEUA when instructed to do so. Four studies noted confusion or lack of understanding about institutional policy regarding informed consent. Students in all 6 studies reported that they believe it is not ethical to perform a PEUA without consent.

### CONCLUSION

While some Students have performed PEUAs without consent, most believe that it is not ethical to do so. Due to the hierarchical nature of medical training, Students reported lack of agency to refuse these exams. Institutional variations in unclear informed consent policies may be challenging for Students to navigate. Limitations of this study include low response rate, inclusion of only English-speaking countries, and bias in self-reported data. Additional studies are much needed due to lack of literature.

